# Individuality That is Unheard of: Systematic Temporal Deviations in Scale Playing Leave an Inaudible Pianistic Fingerprint

**DOI:** 10.3389/fpsyg.2013.00134

**Published:** 2013-03-21

**Authors:** Floris Tijmen Van Vugt, Hans-Christian Jabusch, Eckart Altenmüller

**Affiliations:** ^1^Institute of Music Physiology and Musicians’ Medicine, University of Music, Drama, and MediaHanover, Germany; ^2^Lyon Neuroscience Research Center, CNRS-UMR 5292, INSERM U1028, University Lyon-1Lyon, France; ^3^Institute of Musicians’ Medicine, University of Music “Carl Maria von Weber,”Dresden, Germany

**Keywords:** piano scale, individuality, expertise, music, recognition

## Abstract

Whatever we do, we do it in our own way, and we recognize master artists by small samples of their work. This study investigates individuality of temporal deviations in musical scales in pianists in the absence of deliberate expressive intention. Note-by-note timing deviations away from regularity form a remarkably consistent “pianistic fingerprint.” First, eight professional pianists played C-major scales in two sessions, separated by 15 min. Euclidian distances between deviation traces originating from different pianists were reliably larger than traces originating from the same pianist. As a result, a simple classifier that matched deviation traces by minimizing their distance was able to recognize each pianist with 100% accuracy. Furthermore, within each pianist, fingerprints produced by the same movements were more similar than fingerprints resulting in the same scale sound. This allowed us to conclude that the fingerprints are mostly neuromuscular rather than intentional or expressive in nature. However, human listeners were not able to distinguish the temporal fingerprints by ear. Next, 18 pianists played C-major scales on a normal or muted piano. Recognition rates ranged from 83 to 100%, further supporting the view that auditory feedback is not implicated in the creation of the temporal signature. Finally, 20 pianists were recognized 20 months later at above chance level, showing signature effects to be long lasting. Our results indicate that even non-expressive playing of scales reveals consistent, partially effector-unspecific, but inaudible inter-individual differences. We suggest that machine learning studies into individuality in performance will need to take into account unintentional but consistent variability below the perceptual threshold.

## Introduction

Our actions are highly individual and we can tell people apart by how they move (Flach et al., 2004; Loula et al., 2005; Prasad and Shiffrar, 2009; Sevdalis and Keller, 2011). People may recognize those close to them by the way they sneeze or walk the stairs. Even when trying to achieve the same aim, the actions that are selected toward this aim and the way in which they are executed vary considerably between individuals. The human observer seems to rely on action simulation to recognize individuals by their movements, since recognition is generally stronger when distinguishing one’s own performance from that of others (Jeannerod, 2003).

A first question is how movements from different individuals vary *physically*. Why are certain parameters of our actions remarkably stable between multiple iterations by the same person, and yet strikingly different between individuals? A second question is to what extent movements vary *perceptually*. For example, some movements may differ so subtly that the individual features are not distinguishable to a human observer under normal conditions.

Music is a suitable paradigm to study individuality since actions are directed toward a clearly defined auditory goal: when we play music, the aim is to make a certain sound. Furthermore, differences between performers are sometimes so salient that listeners will often refuse to listen to a musical piece that is a mere “cover” of the original. Music played by different individuals varies *physically*. For example, machine ensemble learning approaches are able to tell musical performers apart based on structural features such as timing and loudness differences (Stamatatos and Widmer, 2005) or kinematics (Dalla Bella and Palmer, 2011). The individuality is also *perceptual*. Indeed, non-musicians and musicians alike were able to recognize performances reliably (Gingras et al., 2011). Again, action simulation in the form of musical imagery appears to play a role in the recognition process. For example, piano players turn out to be capable of recognizing their own playing from a few months previously, even if the sound was switched off at the time of the recording (Repp and Knoblich, 2004).

In music performance recognition the differences in sound that different players produce are often understood as a result of their artistic individuality. However, there is no reason to assume that the individuality in the way we walk serves any particular purpose. Indeed, even task-irrelevant sounds matching a golf swing are recognized significantly better than chance (Murgia et al., 2012). On the other hand, individuality in music performance is tacitly assumed to define a performer’s unique artistic identity. But we have to date no empirical validation of the extent to which individuality in music performance is deliberate. The study coming closest to answering this question requested pianists to play mechanically, and found that recognition was somewhat impaired for these inexpressive recordings (Gingras et al., 2011). However, even metronomic playing has been shown to contain the same timing patterns as expressive playing, but to a lesser extent (Repp, 1999a). To avoid this problem, we instead investigated the playing of musical scales (Wagner, 1971; MacKenzie and Van Eerd, 1990). When participants are instructed to play a scale as regularly as possible and in a legato style, there is a clear auditory target of perceptual evenness and it is understood that the task at hand is not to play scales in one’s own particular way. In other words, isolated scales are not thought of as expressive musical materials. There is some objective standard and trying to meet it is a merely technical task.

Yet, it is found that musical scales show systematic temporal deviations (MacKenzie and Van Eerd, 1990; van Vugt et al., 2012). These deviations are thought of as the result of perceptual distortions (Drake, 1993), residual expressive timing (Repp, 1999a), or of some note transitions involving more difficult movements (Engel et al., 1997).

Our question is whether these temporal deviations are individual in the same way that expressive performance is. We restrict our attention to timing of note onsets, discarding information such as differences in loudness and note duration. In Experiment I, we first established timing deviations of individual notes (van Vugt et al., 2012). The resulting timing profile is then used to recognize pianists across two sessions, separated by 15 min. In this way, we aim to establish individuality that is physically present in the timing of musical scales. In Experiment II, we then proceed to assess whether the timing differences can be perceived by musically trained observers. In Experiment III we investigate the role of auditory feedback in the formation of these timing profiles. Finally, in order to investigate to what extent these timing deviation profiles are stable, we follow a group of pianists over 27 months in Experiment IV.

## Experiment I

### Materials and methods

The data reported here were collected as part of a validation procedure for a scale unevenness quantification method published elsewhere (Jabusch et al., 2004). Eight pianists (six female) were recruited from the student/teacher pool at the Hanover University of Music and were 24.3 (SD 2.4) years old. All but one were right-handed (*M* = 57.2, SD = 66% right-handed according to the Edinburgh handedness inventory). None of the participants reported any neurological condition. Participants played on a MP 9000 MIDI keyboard (Kawai, Krefeld, Germany). The keyboard’s digital music interface (MIDI out) signal was captured on a PC using a commercially available sequencer software (Musicator Win, version 2.12; Music Interactive Technology, Bergen, Norway).

Participants were requested to play two-octave C-major scales beginning with the C (131 Hz) one octave below the middle C and ending with the C (523 Hz) one octave higher than the middle C. Ascending and descending scales were interleaved. The instruction to the participants was to play as evenly as possible, without expression, and in a legato style at mezzo-forte loudness. A metronome gave a beat at 120 BPM and the instructions were to play at four notes per metronome beat, resulting in eight notes per second. Participants performed 10–15 scales with the right hand and with the left hand (*first measurement*). After a 15 min break, the procedure was repeated (*follow-up*).

### Analysis of scale timing

**Figure 1:**
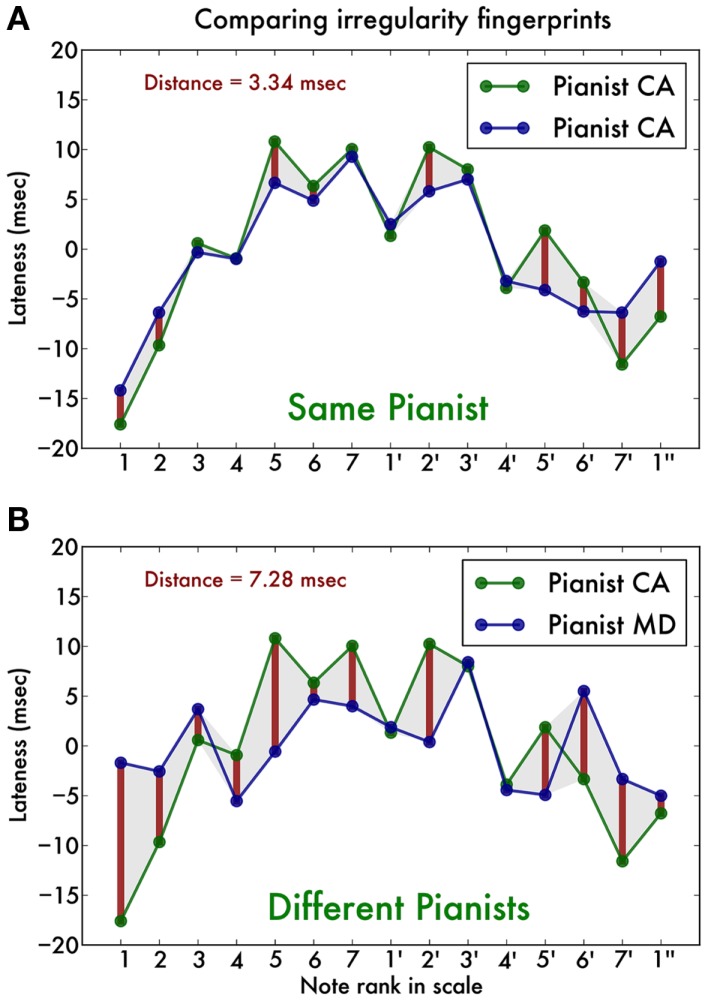
**Illustration of the note onset timing traces of two typical pianists, showing only the right hand ascending scale timings**. One pianist (CA) was recorded playing two-octave C-major scales. Using a previously established technique, we are able to determine the precise timing of each individual note (for further details see text). **(A)** The note-by-note temporal deviation (in ms) is strikingly similar between the two recordings (blue and green line). The red vertical bars and shaded area indicate the temporal distance between the traces, which is on average around 3 ms. **(B)** Comparison of CA’s temporal deviation trace with that of a different pianist (MD). The traces are qualitatively different, which is captured by a higher temporal distance of around 7 ms.

First, we isolated correctly performed scale runs, discarding those containing errors or surplus notes. We then converted the note values to their rank in the C-major scale (i.e., C has rank 0, D has rank 1, E has rank 2, etc., up to C″ with rank 14) and performed a least-square straight line fit to this set of pairs of rank and timing. This allowed us to compute for each note the expected onset time (according to this fit) and then the deviation of the timing of the actually measured onset (in ms) (van Vugt et al., 2012). We performed this fit for all scale runs and then pooled the results by hand (left or right), playing direction (inward or outward) and note, calculating the mean lateness (in ms) for that condition. The result was a 2 (hands) × 2 (directions) × 15 (notes) matrix of timing deviations, which we will refer to as our irregularity trace. As an illustration, Figure 1A shows the irregularity trace for right hand ascending scales in one pianist in the two measurement sessions, and Figure 1B for two different pianists. It is clear that the irregularity traces originating from the same pianist (Figure 1A) are strikingly similar, whereas those originating from different pianists (Figure 1B) are qualitatively different. This is the observation that our analysis (described below) aims to capture.

Additionally, we calculated the unevenness of the scale in accordance with a previously established protocol (Jabusch et al., 2004) as follows. For each correct scale run, the intervals between the consecutive note onsets were calculated and then we took the standard deviation of these. For each hand, direction, and recording (first or follow-up) we took the median of the standard deviations of the scale runs (in ms). The higher this unevenness score, the more temporally irregular the scales.

In ANOVAs we report ${\text{η}}_{G}^{2}$ as the generalized effect size (Bakeman, 2005). Following musicological notational convention, we will refer to the notes in the scale as 1, 2, 3, 4, 5, 6, 7, 1′, 2′, 3′, 4′, 5′, 6′, 7′,1 ″, in ascending order.

### Results

#### Preliminaries

First, we isolated the correctly played scales, yielding an average total of 11.7 (SD 0.97) scales per person and condition. As a control analysis, we used the number of scales as an outcome measurement in an ANOVA that revealed no significant difference according to hand [*F*(1, 7) = 3.43, *p* = 0.11], direction [*F*(1, 7) ≈ 0.00, *p* ≈ 1.00], recording session [*F*(1, 7) = 1.19, *p* = 0.74] nor any interaction effect [all *F*(1, 7) < 0.11]. We can conclude that there is no selection bias due to the discarding of scales.

Now we turn to the unevenness measure (the standard deviation of the inter-keystroke-intervals). ANOVA yielded a significant main effect of hand [*F*(1, 7) = 5.73, *p* < 0.05, ${\text{η}}_{G}^{2}$ = 0.04], showing that left hand scales were played more unevenly (mean unevenness 9.19 ms, SD 1.67) than right hand scales (mean unevenness 8.44 ms, SD 1.81). This replicates a previous finding (Kopiez et al., 2011). There was no main effect of playing direction [*F*(1, 7) = 0.01, *p* = 0.92] nor of recording session [*F*(1, 7) = 1.00, *p* = 0.35] but there was a two-way interaction between direction and recording [*F*(1, 7) = 7.00, *p* = 0.03, ${\text{η}}_{G}^{2}$ = 0.02], showing that although outward scales were played equally evenly across the sessions, inward scales were more even in the follow-up session (unevenness 8.43 ms, SD 1.86) than in the first session (unevenness 9.13 ms, SD 2.33), perhaps revealing a habituation effect.

#### Recognizing individual pianists

A salient feature of the temporal traces is that they are highly individual: traces from the same individual but different sessions vary little, whereas traces from different pianists vary much more (Figure 1). To quantify this observation, we define the temporal distance as the Euclidian distance between any pair of vectors representing the irregularity traces. That is, we calculated the sum of squares of the item-by-item distances. Then we divided this by the number of notes in the traces (15 notes for a two-octave scale). Finally, we took the square root to yield a distance value in ms. First we calculate these distances for each of the two hands, two directions separately. We find that irregularity traces originating from the same pianist have a distance of 3.42 ms (SD 0.89), whereas those originating from different pianists have a distance of 7.24 ms (SD 0.54) (Figure 4). ANOVA with distance as dependent variable shows a significant main effect of self vs. other [*F*(1, 7) = 108.18, *p* < 0.001, ${\text{η}}_{G}^{2}$ = 0.79] but no effect of hand [*F*(1, 7) = 0.55, *p* = 0.48] nor playing direction [*F*(1, 7) = 0.30, *p* = 0.60] nor any interaction effect [all *F*(1, 7) < 1.1].

As a result, we designed the simplest possible classification algorithm as follows. Our algorithm is given a database of the irregularity traces for the first measurements of each of the eight pianists. Then it is presented each of the follow-up irregularity traces, without the player label, and its task is to match each pianist to one of the traces in its database. Our algorithm simply chooses the irregularity trace that matches most closely.

This procedure is performed separately for the four sets of average irregularity traces from the two hands and two playing directions. Classification was flawless (100%) for all the right hand scales (inward and outward), as well as the left hand outward scales. In the left hand inward scales, six pianists are classified correctly and two incorrectly. Chance is at 0.125 recognition rate, meaning that in all cases classification is significantly better than chance [binomial *p* < 0.001, 95% confidence interval = (0.35, 0.97) for the left hand inward scales and (0.63, 1.0) for the other cases]. When instead of the complete irregularity trace (15 data points per two-octave scale) we used only the unevenness (one data point per two-octave scale) classification rate dropped to between 0.25 and 0.5, which exceeded chance performance only for the right hand inward scales [binomial *p* = 0.01, 95% confidence interval = (0.16, 0.84)].

The Euclidian distance is not necessarily the only or best way to quantify the (dis)similarity between irregularity traces. To illustrate this, we perform the same analysis, but this time we compute the correlation (Pearson *r*) between pairs of irregularity traces. ANOVA on the Fisher *r*-to-*z* transformed correlation coefficients shows a main effect of self vs. other [*F*(1, 7) = 63.92, *p* < 0.001, ${\text{η}}_{G}^{2}$ = 0.74], showing that correlations between irregularity traces from the same pianists are higher [*z*(*r*) = 1.39, SD 0.42] than irregularity traces from different pianists [*z*(*r*) = 0.40, SD 0.21]. There is no effect of hand except for a trend [*F*(1, 7) = 5.40, *p* = 0.05, ${\text{η}}_{G}^{2}$ = 0.03], nor a main effect of direction [*F*(1, 7) = 2.76, *p* = 0.14]. Of the interaction effects only that between hand and direction [*F*(1, 7) = 11.50, *p* = 0.01, ${\text{η}}_{G}^{2}$ = 0.10] is significant [all other *F*(1, 7) < 1.05], revealing that whereas left hand traces correlate equally in both playing directions, right hand inward scales correlate higher than outward scales.

We re-ran our recognition algorithm with the only difference that this time, given an irregularity trace to recognize, it chose the irregularity trace that showed the greatest correlation. Recognition rates are identical to those for Euclidian distance: flawless in all but the case of left hand inward scales with six out of two correctly classified (hence still exceeding chance performance).

#### Comparing irregularity traces of the same pianist

**Figure 2:**
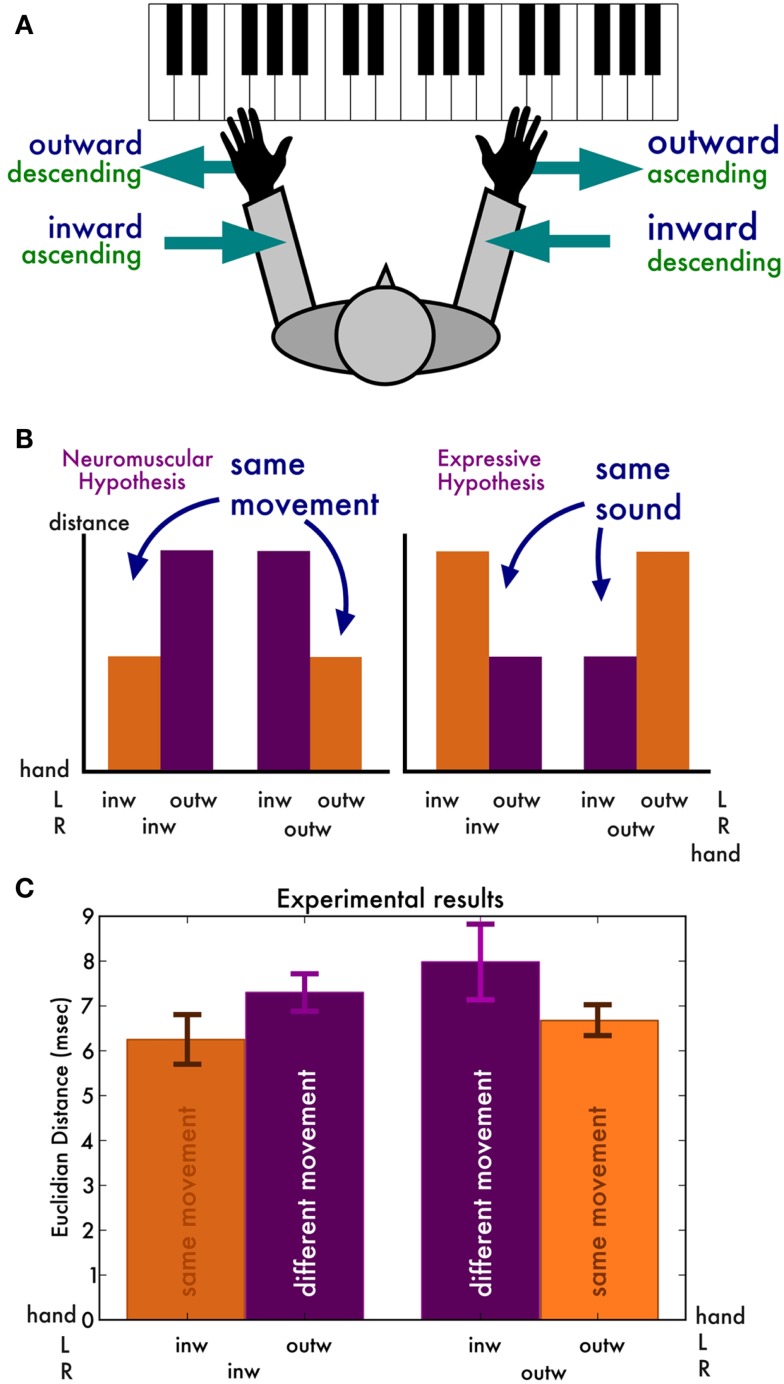
**(A)** Overview of the body-central directions (inward and outward, in blue) and the keyboard-central directions (ascending and descending, in green). **(B)** Predictions of the two hypotheses. If the irregularity traces mostly stem from neuromuscular constraints, we expect traces originating from the same movements to be similar. If they originate mostly from residual expression, we expect traces producing the same sounds to be similar. **(C)** Experimental results, in line with the neuromuscular hypothesis.

So far, we have only compared the irregularity traces produced by the same hand and in the same playing direction but by different pianists. How do the traces produced by the same pianist but by different hands and different directions compare? We argue that these comparisons may provide crucial insight into what causes the timing deviations (Figure 2A). Our reasoning was as follows. If the temporal deviations result from remnants of expressive timing (Repp, 1999a), then we expect irregularity traces that sound similar to be more similar. That is, we expect the left hand inward and right hand outward traces to be closest together (since they have the same auditory result, modulo octave differences), and similarly the right hand outward and left hand inward scales to be close. If, on the other hand, the temporal deviation traces are mostly determined by biomechanical or neuromuscular factors, then we expect traces generated by the same movements to be closer together than those generated by different movements (Figure 2B). More specifically, the pairs of inward and pairs of outward scales are expected to be closer together than pairs with an inward and outward scale.

Furthermore, note that in all these comparisons we have aligned the irregularity traces in time (in the order in which they are played) and not in space (the order in which they appear on the keyboard). That means, when we compare left hand inward and right hand outward scales, they are the same movement in time, but mirrored in space.

An ANOVA with distance as dependent measure revealed a main effect of movement [*F*(1, 7) = 7.63, *p* = 0.03, ${\text{η}}_{G}^{2}$ = 0.10], reflecting that distances between irregularity traces produced by the same movement are shorter (6.47 ms, SD 0.85) than those produced by different movements (7.64 ms, SD 1.58) (Figure 2C). That is, the results are in line with the hypothesis that the temporal deviations are mostly neuromuscular in nature. No other factor has a main effect [all *F*(1, 7) < 1.6] and there were no interactions [all *F*(1, 7) < 2.0].

#### Effector-specificity of the individuality

To what extent is the individuality in the traces specific to the effector (i.e., hand)? To answer this question, we repeated the analysis above, but comparing the distances across hands within and between pianists. That is, we computed the distance between left and right hand irregularity traces for the same movement direction (inward or outward) and for either the same pianist or different pianists. We found a main effect of same vs. different pianist [*F*(1, 7) = 28.35, *p* = 0.001, ${\text{η}}_{G}^{2}$ = 0.01], revealing that cross-hand distances are smaller between traces from the same pianist (*M* = 6.41, SD = 0.87 ms) than traces from different pianists (*M* = 7.47, SD = 0.42 ms). There were no main effects of hand, direction or recording, nor any interaction effects [all *F*(1, 7) < 2.74, *p* > 0.14].

### Discussion

Let us pause an instant to take stock. We have shown that pianists do not play scales perfectly regularly. Rather, consistent temporal deviations are present. For the first time we show that these deviations are not mere noise, since they are reliably reproduced across two recording sessions. Furthermore, differences between individuals are so pronounced that a surprisingly simple recognition algorithm is able to recognize pianists nearly flawlessly using the average timing profile of a dozen runs of two-octave scales. The algorithm works equally well when it matches irregularity traces by minimizing distance or by maximizing correlation.

An important observation is that the pianists’ temporal irregularities are *qualitatively* different. If the irregularity profiles had been qualitatively the same, that is, the same vector simply multiplied by a coefficient, then recognition on the overall unevenness would perform as well as recognition using the entire irregularity trace. But we find the contrary: recognition using a simple overall unevenness metric (the median of the inter-keystroke-intervals) was barely above chance. We can conclude that it is the qualitative differences in the scale timing that enable us to tell the different pianists apart. Hence we can speak of a *pianistic fingerprint*.

What determines this temporal fingerprint? We showed that temporal irregularity traces generated by the same movement are more similar than those generating the same sound. As a consequence, the contribution of biomechanical constraints to these timing profiles must be stronger than expressive or perceptual influences. Furthermore, we found that the individuality in the traces is to some extent effector-independent: the two hands of the same pianist are less different than hands of different pianists. This suggests that the individuality is represented in cortical areas accessible to both effectors (Rijntjes et al., 1999).

In sum, temporal differences are physically present in the produced timing in musical scales. At this point, it remains unclear whether this individuality is also *perceptually* present: are human observers able to identify performers in the same way our algorithm could?

## Experiment II

### Materials and methods

Our perceptual experiment comprised two parts. In the first part (*recognition*), listeners (see details below) were presented with pairs of fingerprint recordings and asked to judge whether they originated from the same or different pianists. Essentially, participants were given the same task that our algorithm in Experiment I performed. In the second part (*irregularity threshold*), we investigated whether participants were able to pick up the temporal irregularities at all by establishing their psychophysical threshold for temporal irregularity. That is, participants were presented a single scale and had to judge whether it was regular (isochronous) or irregular.

#### Recognition test

We took the irregularity traces for the right hand ascending scales for three pianists (CA, ES, and TY) from the *first* and *follow-up* measurements in Experiment I. For each, we furthermore choose one alternative pianist from the *follow-up* measurements (MD, IM, and VH, respectively). Each stimulus consists of a pair of scales played one after the other. These six scale pairs are listed in Table 1. Participants responded by pressing a button whether they felt the two scales were played by the same pianist or different pianists.

**Table 1:** **Stimuli for the recognition experiment**.

Pianist (*first*)	Pianist (*follow-up*)	Comparison	Fingerprint distance (ms)	SD-IKI *first* (ms)	SD-IKI *follow-up* (ms)
CA	CA	Self	3.34	7.78	5.08
CA	MD	Other	7.28	7.78	6.65
ES	ES	Self	3.38	8.85	9.35
ES	IM	Other	8.30	8.85	8.39
TY	TY	Self	3.37	7.29	7.42
TY	VH	Other	7.34	7.29	9.22

*SD-IKI is the Standard Deviation of the inter-keystroke-intervals (in ms)*.

The two scales in a pair were played preceded by two high-pitched notes (MIDI note 96), providing a tempo reference at 120 BPM. The scales were then played with four notes per metronome click, that is, at eight notes per second. The second scale always started 3.5 s after the first. All notes had a duration of 137.5 ms to generate legato style and a standardized loudness level. That is, we removed all loudness cues as well as articulation. Furthermore, each scale pair came in two versions: a *veridical* rendition, and a *magnified* rendition where all timing deviations were increased by a factor 5 (for a similar strategy in the context of a recognition experiment, see Hill and Pollick, 2000). In other words, we multiplied the irregularity vector by a scalar, making the differences more salient. The six stimuli (Table 1) were rendered twice (veridical and magnified), and presented in the two possible orderings, yielding 24 stimuli. Each of these were presented six times, yielding a total of 144 stimuli. The order was randomized for each participant and divided into 4 blocks of 36 trials.

For data analysis, we used the R Package for Statistical Computing and the signal detection scripts developed by Prof. Abby Kaplan (http://home.utah.edu/~u0703432/).

#### Irregularity threshold test

We extracted the irregularity traces of the right hand ascending scales for three pianists (CA, ES, and MD). The irregularity vector was multiplied by a scalar *factor* (between 0 and 5) and was then written as a MIDI file with eight notes per second, preceded by two metronome clicks at 120 BPM. For example, a factor of 0 means a perfectly regular (i.e., isochronous) scale, a factor of 1 corresponds to the scale as it was played in actuality, and a factor of 5 means that all note timings are five times more early or late than they were in reality whilst keeping the overall tempo intact. Participants were asked to report whether the scale sounded regular or irregular.

We used the maximum likelihood procedure (MLP) (Green, 1993; Gu and Green, 1994) to detect the threshold of the factor variable. Participants performed three thresholding blocks, one for each of the sample fingerprints. At the beginning of each block, we deployed 500 hypothetical psychometric curves with their midpoints linearly spaced over the factor levels from 0 to 5, crossed by the five false alarm rates of 0, 10, 20, 30, and 40%, yielding a total of 2,500 hypothetical psychometric curves maintained online in parallel. The slope parameter of these curves was set to four, since no prior experimental data exists and the slope has been shown not to influence the resulting thresholds all that much (Gu and Green, 1994). This yielded the following equation for the psychometric curves: *p*(yes) = *a* + (1−*a*) × (1/(1 + exp[−*k* × (*x* − *m*)])), where *x* is the stimulus level (i.e., the factor), *a* is the false alarm rate, *m* is the mean of the psychometric curve, *k* is the slope parameter (4), *p*(yes) is the probability of responding “irregular.”

Each block consisted of 36 trials. On each trial, we calculated online the likelihood of the set of previous participant responses for each of the 2,500 hypothetical psychometric curves. The curve with the maximum likelihood was chosen as the current estimate. The magnification factor for that given trial was determined by the 64%-response point of this current estimate psychometric curve. In this way, the algorithm is shown to converge rapidly to the participant’s threshold (Green, 1993). We furthermore inserted two catch trials (with factor level 0 regardless of the current psychometric curve estimate) the first 12 trials at random locations, as well as four more over the remaining 24 trials.

Stimuli were written as MIDI files and then played through Timidity++ on a Windows computer, called by our Python (Pygame) graphical interface that registered the responses. The MLP computation was implemented in PythonMLP (which we have made available open-source online at: https://github.com/florisvanvugt/PythonMLP).

#### Participants

Ten pianists from the Hanover University of Music student pool participated in this perceptual experiment. Participants (four female) were 24.8 (SD 3.7) years old and studied piano as their primary instrument. Further, they had normal hearing and reported no neurological impairments. The experiment took approximately half an hour and participants received a nominal payment for their participation.

### Results

#### Recognition test

**Figure 3:**
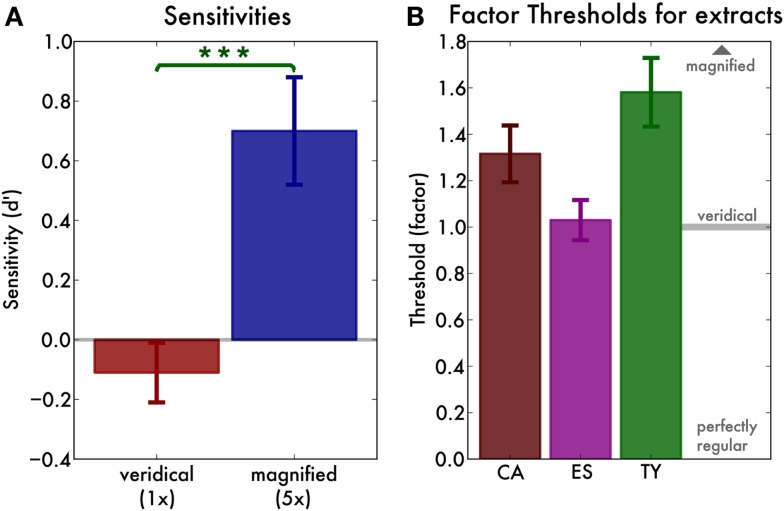
**(A)** Main effect of factor (veridical or magnified) in the recognition experiment. Sensitivity (*d*′) is not greater than zero for the veridical rendering (factor 1), but is greater than zero for the magnified (factor 5) rendering. The error bars indicate the standard error of the mean. **(B)** Irregularity thresholds for three representative fingerprints. We find that the thresholds for all three extracts are one or above, that is, their irregularity is heard only when we exaggerate it slightly. Error bars indicate the standard error of the mean.

We used signal detection theory to calculate sensitivity (*d*′) for the individual participants, fingerprint pairs, and the factors (veridical or magnified) separately. There was a main effect of factor [*F*(1, 9) = 10.84, *p* = 0.001, ${\text{η}}_{G}^{2}$ = 0.25], reflecting that sensitivity was greater for magnified (mean *d*′ = 0.70, SD = 0.58) than for veridical (mean *d*′ = −0.11, SD = 0.31) pairs (Figure 3A). There was no main effect of fingerprint pair [*F*(2, 18) = 1.44, *p* > 0.2] but there was an interaction between factor and fingerprint pair [*F*(2, 18) = 6.09, *p* < 0.01, ${\text{η}}_{G}^{2}$ = 0.23]. As a result, we investigated the sensitivity for each extract separately. For the veridical renditions, none of the sensitivities significantly exceeded zero [all *t*(9) < 0.7, *p* > 0.25], indicating that participants were not able to distinguish pairs of recordings from the same pianist from pairs from different pianists. However, for the magnified renditions of the CA-MD and ES-IM pairs, sensitivity was significantly above zero [*t*(9) = 2.79, *p* = 0.01, and *t*(9) = 3.85, *p* < 0.01, respectively]. Only for the magnified TY-VH pair participants’ sensitivity was zero [*t*(9) = 0.58, *p* = 0.29].

After completing all blocks in this part of the experiment, participants were asked to subjectively rate the confidence in their answers on a five-point Likert scale from very confident (1) to very unsure (5). For the magnified fingerprint pairs, participants were mildly confident (median 3.5, range 2–4). For the veridical pairs, participants were similarly confident (median 4, range 3–5). The ratings did not differ significantly (Mann–Whitney *U* = 10.5, *p* = 0.29). We can conclude that although participants performed much better in the magnified pairs, they were not aware of this improvement in performance.

#### Irregularity threshold test

We discarded blocks in which participants’ “irregular” response ratio for the catch trials exceeded 30%. This was the case for one block of one participant. The threshold for the remaining blocks was defined as the midpoint of the maximum likelihood estimate psychometric curve. Overall, curve midpoints expressed as factor were around or slightly above one (Figure 3B), meaning that the irregularities became audible only when they were slightly increased (factor >1). The thresholds were entered into a one way ANOVA with fingerprint (the three example fingerprints) as a factor. There was a main effect of fingerprint [*F*(2, 26) = 4.85, *p* = 0.02, ${\text{η}}_{G}^{2}$ = 0.27], indicating that the threshold factors were different for the different extracts. However, the fingerprints differed in evenness at the outset (see Table 1). As a result, we expressed the threshold not as a factor but as the corresponding unevenness value (SD of the inter-keystroke-intervals). We then re-ran the ANOVA and found no main effect of extract [*F*(2, 26) = 1.58, *p* = 0.22]. The average threshold unevenness threshold value was 10.22 ms (SD 2.51).

### Discussion

From our threshold experiment, we can conclude that the thresholds straddle the boundary of the timings as actually played (i.e., slightly above factor 1). Our interpretation is that pianists train to make their scale playing more regular until the irregularities are no longer audible.

We conclude that participants are not able to tell the difference between a scale as played by a pianist and an isochronous scale. It naturally follows that they will then not be able to differentiate between pianists since both scales sound regular (isochronous) to them. Indeed, in our recognition test participants were unable to distinguish pairs of scales played by the same pianist from pairs played by different pianists. However, when we magnified the timing deviations by a factor of five, the participants performed above chance in the recognition task. This shows that, in principle, the task of distinguishing scale playing of one pianist from another can be done. These two tests, taken together, constitute evidence that participants were not able to hear the differences between the pianist fingerprints and categorize them on the basis of these differences.

Our study is also the first to systematically investigate thresholds for perception of irregularity in piano scales. We find that the irregularities in recorded piano scales are slightly below the perceptual threshold. This in itself is an interesting finding. Our interpretation is that pianists practice to make their scale playing sound regular but do not continue to make it more regular once it is below the perceptual threshold. For one, listeners will not be able to tell the difference, and secondly, if the motor learning of scale regularity is guided by auditory feedback (Jäncke, 2012) only, they will not be able to improve their temporal regularity once they fall below the auditory threshold.

We furthermore found that the differences in threshold between the extracts can be explained by their difference in unevenness: more temporally uneven fingerprints have a lower factor threshold, whereas more temporally even fingerprints have a higher threshold. This suggests that the obtained threshold of 10.22 ms is independent of the particular temporal fingerprint. We conclude that the unevenness captures the auditory percept of unevenness and no more complex auditory gestalt needs to be taken into account to explain the thresholds. The threshold corresponds to some 8.2% of the interval at this tempo, which is in line with the typical 10% threshold of a single late or early note in an otherwise isochronous sequence (Hyde and Peretz, 2004; Ehrlé and Samson, 2005).

Since these individual characteristics of the scale fingerprints are inaudible, it seems that their production is not dependent on auditory feedback. However, this conclusion is not warranted, since it could be that the timing deviations are residuals of expressive timing (Repp, 1999a). To clarify this issue, we investigated whether the pianistic fingerprints were affected by playing on a mute piano.

## Experiment III

### Materials and methods

Eighteen piano students (nine female) from the Hanover University of Music were invited to play two-octave C-major scales in two recordings. Participants were 28.2 (SD 5.8) years old. In the first recording, participants heard the sounds they produced (*sound*) but in the second recording the sound was switched off (*mute*). In both recordings, scales were played by one hand and then by the other. Otherwise, the procedure and analysis was identical to before. We report 95% confidence intervals (CI) unless otherwise stated.

### Results

We discarded incorrectly played scales leaving a total of 13.4 (SD 1.77) per condition. There was no effect of hand, direction, or recording on the number of correctly produced scales [all *F*(1, 17) < 1.8]. There was a significant but marginally small interaction between hand and direction [*F*(1, 17) = 4.71, *p* = 0.04, ${\text{η}}_{G}^{2}$ = 0.001] and none of the other interactions was significant [all *F*(1, 17) < 4.3].

As before, the distances between fingerprints originating from the same pianist are smaller than those originating from different pianists [*F*(1, 17) = 168.2, *p* < 0.001, ${\text{η}}_{G}^{2}$ = 0.55]. There was a (small) interaction between hand and direction [*F*(1, 17) = 7.45, *p* = 0.01, ${\text{η}}_{G}^{2}$ = 0.03], indicating that for the right hand, inward scales are more similar than outward scales, whereas for the left hand this was the opposite.

Our distance-minimizing algorithm introduced in Experiment I correctly recognized between 8 (44%) and 12 (67%) of the 18 pianists using the fingerprint for only one hand and direction at a time. This exceeds chance performance, which lies at 6%. The correlation-maximizing algorithm correctly recognized between 7 (39%) and 15 (83%) pianists.

When we combined the two hands and two directions (yielding a 2 × 2 × 15 fingerprint matrix for each participant) and perform the same classification, the distance-minimizing algorithm correctly identified 15 out of 18 pianists [83%, binomial *p* < 0.001, confidence interval (0.59, 0.96)]. Crucially, the result is the same whether matching the *mute* fingerprints, one by one, to the set of *sound* fingerprints, or the other way around, indicating that there is no loss of information in the *mute* condition. The correlation-maximizing algorithm also recognizes 15 out of 18 pianists when it finds matching *sound* fingerprints to a given *mute* fingerprint, and the other way around spectacularly recognizes all 18 pianists [100%, binomial *p* < 0.001, CI (0.81, 1.00)].

In order to compare our results with those of Experiment I, we take 10,000 bootstrap samples of eight (unique) pianists and perform the classification with those. The correlation-maximizing algorithm recognizes 95% of pianists [SD 8%, bootstrap CI (75, 100)] whereas the distance-minimizing algorithm recognizes 90% of pianists [SD 8%, bootstrap CI (75, 100)]. That is, they do not perform significantly differently.

### Discussion

It is becoming clear that having auditory feedback while playing the scales is not of importance in the formation of the pianistic fingerprint. Indeed, it is a typical finding in performance literature that absence of auditory feedback only marginally affects performance (Repp, 1999b) or not at all (Gates and Bradshaw, 1974). The findings are furthermore in line with our previous result that fingerprints generated by the same movements are more similar than those generating the same sounds (Experiment I).

Finally, we turn to the question of how stable these fingerprints are over time.

## Experiment IV

### Materials and methods

We re-analyzed data published previously (Jabusch et al., 2009) in which 20 pianists’ (eight female) scale playing was measured twice (first, follow-up) with an interval of 27.8 (SD 8.8) months. At the first measurement, pianists were 27.7 (SD 6.0) years old and had accumulated 21.6 (SD 11.0) thousand hours of lifetime piano practice (not counting one pianist who had not reliably reported this figure). In between the two measurement sessions, pianists accumulated an additional 2.8 (SD 1.8) thousand practice hours, amounting to an average 3.31 (SD 1.79) hours per calendar day (including weekends and holidays). All but two pianists were right-handed according to the Edinburgh handedness inventory (Laterality Quotient: *M* = 73%, SD 56).

### Results

After discarding incorrect scales we were left with 13.5 (SD 0.8) scales of the *first* measurement and 12.8 (SD 1.2) scales at the *follow-up* measurement. This difference was significant [*F*(1, 19) = 5.65 *p* = 0.03 ${\text{η}}_{G}^{2}$ = 0.09].

As before, distance was smaller between recordings of the same pianist than that of different pianists [*F*(1, 19) = 184.90, *p* < 0.001, ${\text{η}}_{G}^{2}$ = 0.30]. Furthermore, distance was generally smaller between fingerprints of the right hand than those of the left hand [*F*(1, 19) = 6.33, *p* = 0.02, ${\text{η}}_{G}^{2}$ = 0.05], perhaps reflecting the greater training of the right hand (Kopiez et al., 2011). For brevity, we only report recognition results using the fingerprint combining both hands and directions. Recognition based on minimizing distance successfully found first recordings given the follow-up fingerprints in 13 pianists [65%, binomial *p* < 0.001, CI (40, 85)%]. Conversely, seven pianists were recognized based on their follow-up measurement [35%, binomial *p* < 0.001, CI (15, 59)%]. Recognition by maximizing correlation performed similarly with 13 (65%) and 8 [40%, binomial *p* < 0.001, CI (19, 64)%] correct identifications.

Bootstrap analysis was performed (see Experiment III) with 10,000 samples of eight pianists. Correlation recognition identified 73% [SD 15%, bootstrap CI (38, 100)%] of pianists and distance recognition 71% [SD 18%, bootstrap CI (38, 100)%]. Based on the bootstrap CI we can see that across the three experiments, identification was equally successful.

How is the stability of a pianist’s fingerprint related to how much he or she practised between the two measurements? We calculated the distance for both hands and playing directions and correlated this to the number of practice hours accumulated between the two measurement points. The distances between the right hand outward scale fingerprints correlated negatively with amount of practice (Pearson *r* = −0.71, *p* = 0.001). That is, those who practised more showed smaller distances between their fingerprints. This does not mean that the fingerprints showed less deviations from regularity, but instead, that the deviations that were present were more consistently reproduced. The right hand inward fingerprints showed a tendency for the same correlation (Pearson *r* = −0.46, *p* = 0.05) but the left hand fingerprints did not (*r* > −0.34, *p* > 0.16).

### Discussion

**Figure 4:**
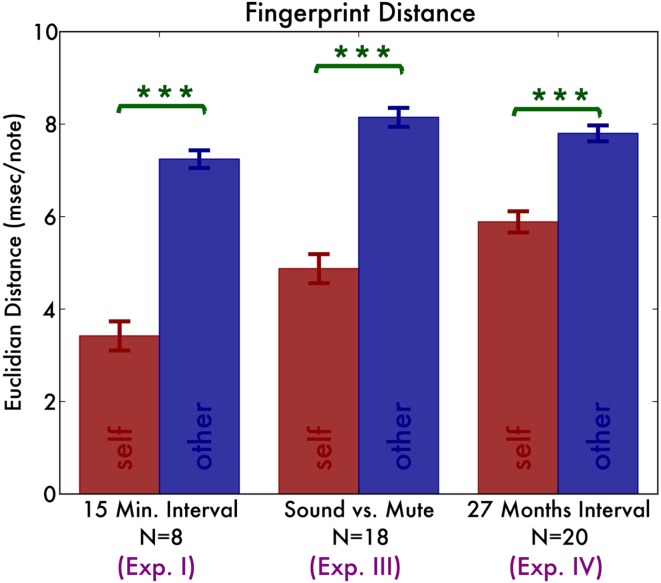
**Summary of the distances between fingerprints originating from the same pianist (*self;* the red bars) and fingerprints originating from different pianists (*other*; the blue bars)**.

**Figure 5:**
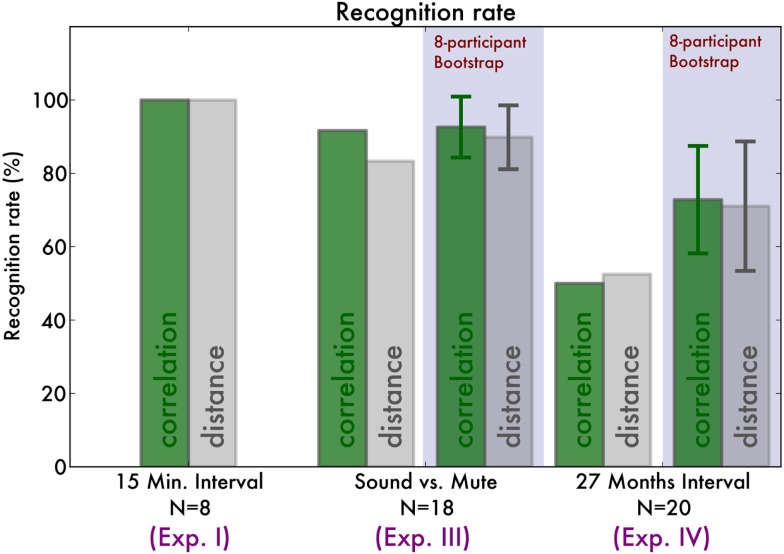
**Overview of the recognition rates of our recognition algorithm**. The green bars indicate the correct classification rate by maximizing fingerprint correlation, and the gray bars by minimizing fingerprint distance. For comparison, we indicate the bootstrap classification results, indicating for each experiment the average recognition rates across eight-pianist bootstrap samples. Error bars indicate the standard deviation of the recognition rates.

The fingerprints that enabled reliable identification of pianists were sufficiently stable to still allow recognition after 27 months. Figure 4 compares the distances across the Experiment I, III, and IV and Figure 5 displays the recognition rates. Although it seems the recognition is worse in Experiment III and IV, the 95% bootstrap CI still include the 100% recognition rate of Experiment I. Therefore we conclude that recognition is not significantly different across the experiments.

## General Discussion

Artists are recognized reliably based on their work (Yamamura et al., 2009). The present study investigated pianist recognition based on non-expressive materials. Taking scale playing as an example, this study brings to light a highly individual temporal signature that enables robust identification of pianists using a simple algorithm. Clearly an individual timing signature is present physically, but perceptual recognition performance by musician listeners was at chance because the deviations were below their perceptual thresholds. Fingerprints appear to stem from neuromuscular factors in the pianists, rather than auditory feedback. This is confirmed in Experiment III that shows fingerprint formation is not affected by absence of sound. The fingerprint is furthermore robust, showing only mild changes in professional pianists over a 27-month interval.

The findings are in line with previous studies showing that pianists can be reliably recognized even when asked to not play expressively (Gingras et al., 2011). Our result strengthens the interpretation that recognition is based on non-expressive clues by employing materials (musical scales) with a clear auditory goal of regularity. Moreover, we have at present only used timing information, discarding loudness and articulation markings that could potentially be used to enhance recognition. The recognition algorithm that we present pairs fingerprints with minimum distance or maximum correlation. The proposed similarity metric is transparent and easy to interpret (see Figure 1). As such, it is surprisingly simple compared with neural networks typically employed (Stamatatos and Widmer, 2005; Dalla Bella and Palmer, 2011).

The idea that artists can be recognized by a non-artistic feature of their work is not new. For example, painters can be automatically recognized by stroke style (Li et al., 2012). Beyond the realm of art, authorship can be established by relatively irrelevant features of produced work. For example, handwriting is highly individual (Rijntjes et al., 1999) and pattern recognition using word frequencies has been employed to establish Madison as the author of the 12 disputed Federalist papers (Mosteller and Wallace, 1964). Similarly, telegraph operators during the Second World War claimed to be able to identify the sender by the timing of his keystrokes (“Fist of sender”). The emerging field of keystroke dynamics puts this to use to authenticate computer users by their typing rhythm instead of through a password (Bergadano et al., 2002). Typically the problem remains that over time these dynamics change and recognition becomes impaired. In light of this, it is interesting that our recognition was highly stable even in a fairly homogeneous sample of expert pianists (Experiment IV). Recognition in keystroke dynamics as well as in our result may be based to some extent on the subunits that the produced sequences are divided into, i.e., its chunking (Sakai et al., 2003). On the other hand, more low-level neuromuscular properties such as the individual anatomy, especially tendon-ligament anatomy or the strengths of the individual muscles are more likely to be at the root of these individual temporal irregularities, since the sequences under consideration here (the scales) are greatly over-learned. Future studies may decide this issue by investigating recognition of pianists playing at various tempi, since although chunking may vary across speeds, the neuromuscular properties will remain constant.

We propose that studies investigating the individuality of artists, especially those employing machine learning strategies (Stamatatos and Widmer, 2005), may take into account that a large part of this individuality is inaudible and merely neuromuscular in nature. In the future, one could tease apart cues that are uniquely expressive and those that are neuromuscular.

Artistic individuality is typically thought to be deliberate and determined by top-down cognition. Our study opens the road to investigation into the tantalizing question of how biomechanical constraints may determine artistic performance in a bottom-up fashion.

## Conflict of Interest Statement

The authors declare that the research was conducted in the absence of any commercial or financial relationships that could be construed as a potential conflict of interest.
